# In Vivo Biodistribution and Toxicity of Highly Soluble PEG-Coated Boron Nitride in Mice

**DOI:** 10.1186/s11671-015-1172-0

**Published:** 2015-12-10

**Authors:** Bo Liu, Wei Qi, Longlong Tian, Zhan Li, Guoying Miao, Wenzhen An, Dan Liu, Jing Lin, Xiaoyong Zhang, Wangsuo Wu

**Affiliations:** Radiochemistry Laboratory, Lanzhou University, Lanzhou, Gansu 730000 China; Huazhong University of Science and Technology, Wuhan, 430074 China; Key Laboratory of Chemistry of Northwestern Plant Resources, Key Laboratory for Natural Medicine of Gansu Province, Institute of Chemical Physics, Chinese Academy of Sciences, Lanzhou, Gansu 730000 China; Department of Radiotherapy, Gansu Provincial Hospital, Lanzhou, Gansu 730000 China; School of Life Sciences, Lanzhou University, Lanzhou, Gansu 730000 China; Department of Chemistry, Nanchang University, 999 Xuefu Avenue, Nanchang, 330031 China

**Keywords:** Biodistribution, Toxicity, Soluble, Boron nitride (BN)

## Abstract

The boron nitride (BN) nanoparticles, as the structural analogues of graphene, are the potential biomedicine materials because of the excellent biocompatibility, but their solubility and biosafety are the biggest obstacle for the clinic application. Here, we first synthesized the highly soluble BN nanoparticles coated by PEG (BN-PEG) with smaller size (~10 nm), then studied their biodistribution in vivo through radioisotope (Tc^99m^O_4_^−^) labeling, and the results showed that BN-PEG nanoparticles mainly accumulated in the liver, lung, and spleen with the less uptake by the brain. Moreover, the pathological changes induced by BN-PEG could be significantly observed in the sections of the liver, lung, spleen, and heart, which can be also supported by the test of biochemical indexes in serum. More importantly, we first observed the biodistribution of BN-PEG in the heart tissues with high toxicity, which would give a warning about the cardiovascular disease, and provide some opportunities for the drug delivery and treatment.

## Background

The two-dimensional (2D) materials are a single-atom-layer materials with some special properties [1–3]. At present, the most famous 2D material is the carbon 2D nanomaterials namely graphene because of its excellent physicochemical properties [4–6]. Moreover, the boron nitrides, known as white graphene [7], have received more and more attention. BNs are structural analogues of graphene in which C atoms are replaced by alternating B and N atoms. Moreover, thanks to the special chemical and physical characteristics of the BN nanoparticles, many researchers have found a large number of applications in the field of nanotechnology, and several studies have shown their possible exploitation in the biomedical domain, such as nanocarriers [8] and nanotransducers [9]. However, before the clinical application, it is very necessary and important for biomaterials of BN to investigate biosafety in vivo and in vitro. Biosafety investigations include toxicity in vivo and cytotoxicity in vitro; to clarify toxicity in vivo, it was essential a prerequisite to research its behavior and fate in the living things. Unfortunately, so far no one has explored the detailed investigations about behavior and toxicity of BN in vivo.

Considering that the pristine BN materials are hardly used in the biomedical domain due to the profound chemical inertness of insoluble BN structures [10], so, a layer of hydrophilic material such as polyethylene glycol (PEG) was often coated on the surface of pristine BN for improving the solubility and enhancing the biological compatibility [11]. Moreover, the PEG that was thought as a safe and innocuous biomaterial is widely used to improve the biological compatibility and water solubility of various nanoparticles [12–14]. Furthermore, the coating of PEG would not alter the structure of BN and remain the property and character of BN, which would increase the application prospects of BN materials [15]. Thereby, Weng et al. [10] successfully prepared the coating of porous BN materials with PEG, which preformed the lower cytotoxicity. However, the cytotoxicity of the porous BN may be different to that of entire BN. Therefore, it would be very imperative to study the behavior and toxicity of entire structure of BN after coating with PEG.

In current works, we found that some PEG-coated BN (BN-PEG) nanoparticles with smaller sizes (~10 nm) and quantum effects could be easily prepared through the treatment of PEG coating, but lower fluorescence quantum yield (~7.8 %) would be not conducive to detect signals from biological samples. Although a fluorescent labeling and imaging may be a popular method to study BN-PEG in vivo, but the potential quenching and other intrinsic limitations of fluorescence imaging could cause nonquantitative and less accurate biodistribution results [10]. In comparison, the pharmacokinetics and biodistribution studies based on ^99m^Tc-labeled, BN-PEG provides more reliable and quantitative information for the in vivo behaviors of biocompatibility functionalized BN [10]. Therefore, here, we prepared the BN-PEG nanoparticles with high water solubility, and then ^99m^TcO_4_^−^ was used to label complexes, and studied the biodistribution and toxicity of BN-PEG in mice in vivo.

## Methods

### Synthesis and Characterization of PEG-Coated BN

A solution of 6-arm-polyethyleneglycol-amine (Sunbio Inc.) (3 mg/mL) was mixed to the BN sheets (0.5 mg/mL) (BN were purchased from Baoding Zhong Pu Rui Ta technology. LTD), and the mixture was stirred at 200 °C for 4 days under a steady nitrogen flow. After cooling the reaction mixture to a room temperature, then H_2_O (~60 mL) was added for extraction [16]. The solution was sonicated and centrifuged (~5000*g*, 20 min), and the supernatants were collected and dialyzed about 1 week by dialysis membrane (MD25) to remove free PEG. Finally, the solution was dried at 50 °C in vacuum oven, and the solid product was the BN-PEG. And then, the product was characterized by TEM (Tecnai G2 F30), XRD (XRD-600) and fluorescence lifetime and steady-state spectroscopy (FLS; 920).

### ^99m^Tc Labeling BN-PEG

**Fig. 1:**
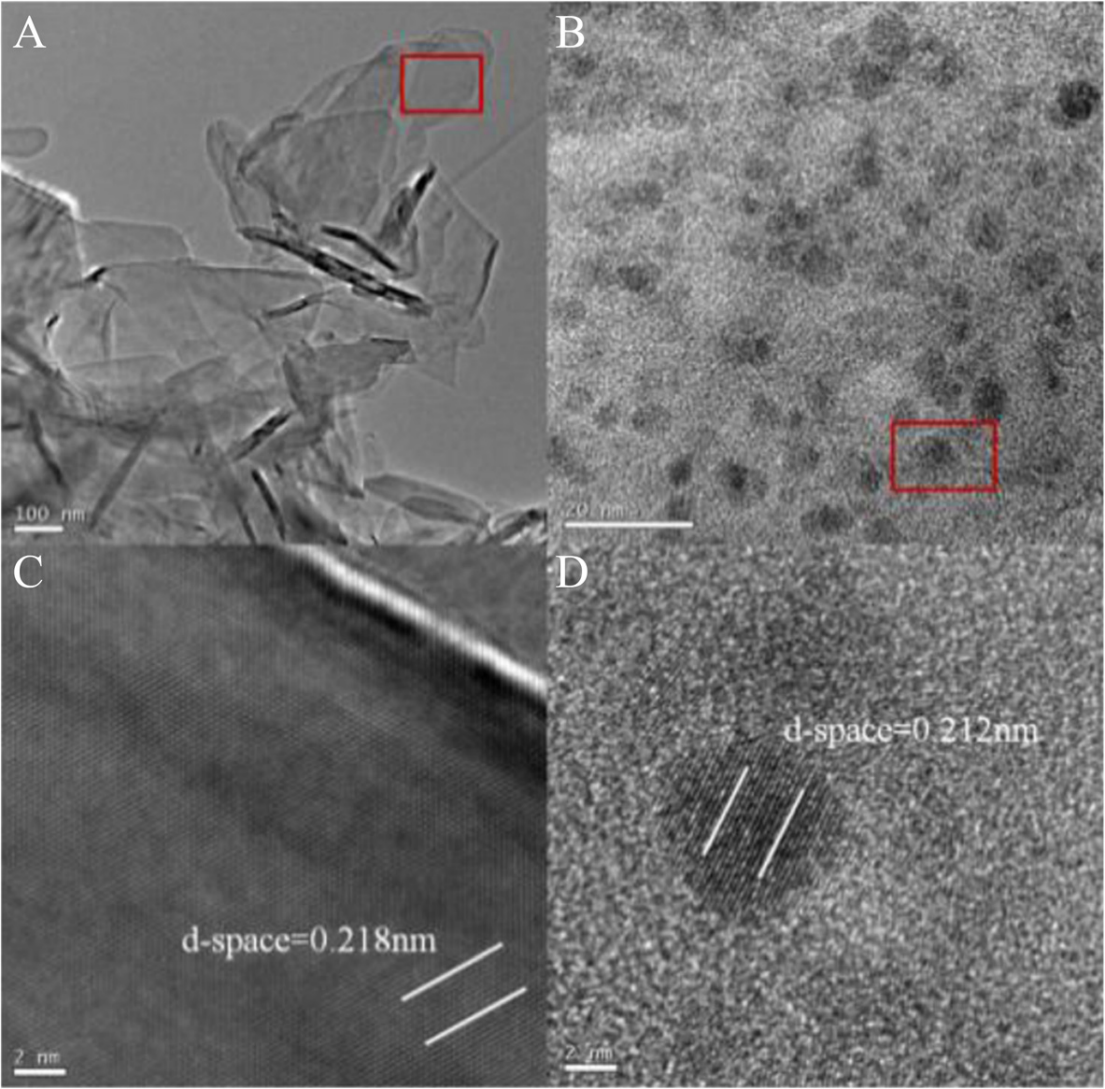
TEM characterization of raw BN (**a**, **c**) and BN-PEG (**b**, **d**); **c** and **d** are the high-resolution optical picture of the limiting region in **a** and **b**, respectively

The ^99m^Tc labeling of BN-PEG and determination of labeling yields were performed [17] (^99m^TcO_4_^−^ was purchased from the China Institute of Atomic Energy, Beijing, China; 5 mCi). BN-PEG was dissolved in deionized water with an ultrasonic device for 30 min. Ascorbic acid, stannous chloride, and ^99m^TcO_4_^−^ were then added into the suspension. This mixture was stirred at 90 °C for 30 min. After centrifugation, the supernatant was decanted, and the remaining solid was identified to be ^99m^Tc-BN-PEG. The radiolabeling yields of ^99m^Tc-BN-PEG were measured by paper chromatography using Whatman 1 paper strips (Maidstone, Kent, UK) (Fig. 1). Considering the short lifetime of ^99m^Tc (6.02 h), we measured the radio counts in 1 day for accurate determination of the tag. Otherwise, the tag (^99m^Tc) would decay in 10 half times.

### The Biodistribution of BN-PEG

Kunming mice initially weighing 15 to 18 g were provided by the Laboratory Centre for Medical Science, Lanzhou University, Gansu, China. All animals were housed in individual cages in a temperature-controlled (21 to 22 °C) and light-controlled (turned on from 0800 h to 2000 h) environment and were fed food and tap water ad libitum. All animal protocols were in accordance with the European Communities Council Directive of November 24, 1986 (86/609/EEC) and approved by Institutional Animal Care and Use Committees of Gansu Province Medical Animal Center and Lanzhou University Animal Committees Guideline (China). The mice were organized randomly into four groups (six mice/group). The mice were injected intravenously with 20 mg/kg bw of ^99m^Tc-BN-PEG solution (pH = 7.26, C_NaCl_ = 0.9 %), and then killed at 1, 6, 16, and 24 h after injection. Tissues from the heart, lung, liver, spleen, kidney, stomach, and brain were immediately dissected, and the blood was also collected. Each tissue was wrapped in foil, weighed and counted for ^99m^Tc. Data were corrected for physical decay of radioactivity. The distribution of the tissues was presented in percent injected dose per gram of wet tissue (%ID/g), which could be calculated by the percent injected dose (tissue activity/total activity dose) per gram of the wet tissue.

### Blood Analysis and Histology Examinations

CRP, ANP, BUN, and TB kits were purchased from Shanghai Heng Hitter Trade Co., Ltd. and then kept in the refrigerator at 4 °C. Approximately 0.2 mL of BN-PEG (20 mg/kg bw) was injected intravenously to the experimental mice (six mice). Approximately 0.2 mL of saline solution and PEG (20 mg/kg bw) was administered to the mice from the control groups. All mice were then killed. Approximately 2 mL of blood was collected to obtain the plasma, and then centrifuged for 10 min to collect the serum. The serum contents of CRP, ANP, BUN, and TB were then measured. At the same time, the liver, lung, spleen, kidney, and heart were harvested immediately. These tissues were fixed in 10 % buffered formalin and processed for routine histology with hematoxylin and eosin staining by the Centre for Medical Science, Lanzhou University (Lanzhou, China). Microscopic observation of tissues was performed with an Olympus Microphot-CX41 microscope coupled with a digital camera.

## Results and Discussion

### The Preparation and Characterization of BN-PEG

**Fig. 2:**
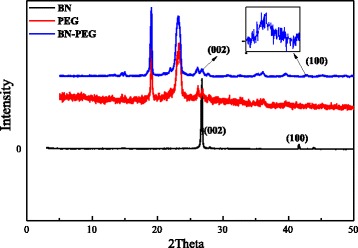
XRD characterization of raw BN, PEG, and BN-PEG (the lattice spacing for (002) is 0.33 nm and for (100) is 0.217 nm)

**Fig. 3:**
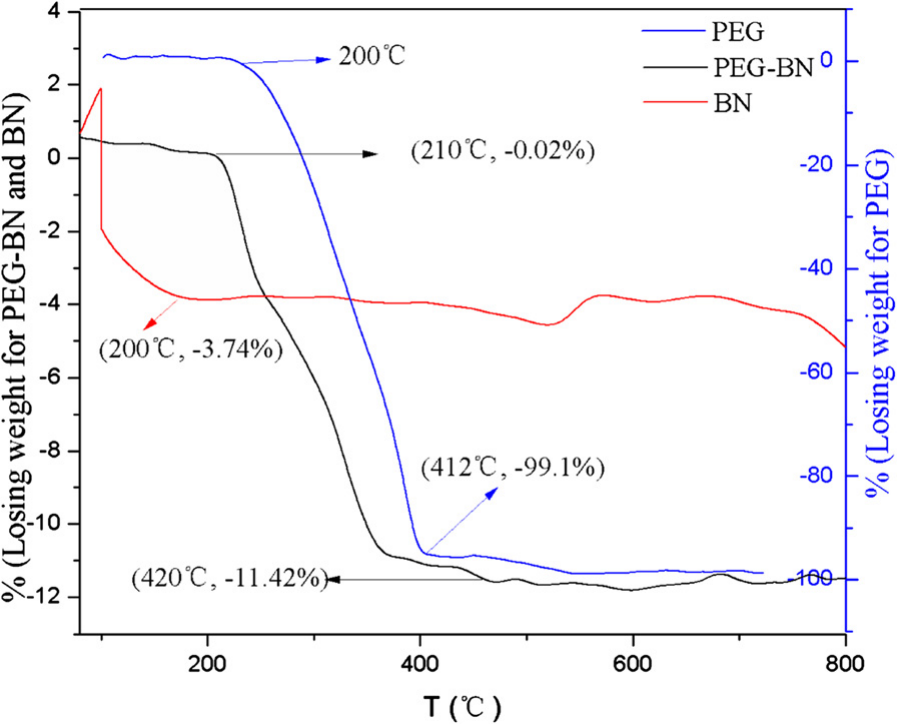
TGA of BN, PEG, BN-PEG (the samples were made under protection N_2_ with a flow speed about 0.06 L/min)

**Fig. 4:**
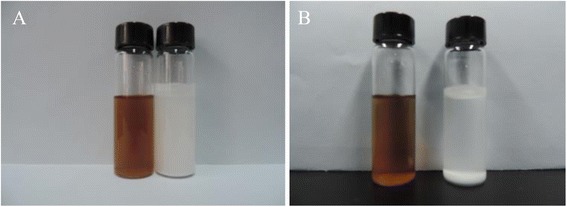
The study of water-soluble in 0.5 min and 24 h, **a** 0.5 min; **b** 24 h. The brown is the BN-PEG; the white is the pristine BN

The Fig. 1 showed the image of raw BN and BN-PEG from the TEM, which showed that the morphology of raw BN is very disordered. After coating with PEG, the size of BN-PEG became relatively uniform and smooth with clear stripe structure of 2D, which may be due to that bigger size of BN was removed in the process of preparation. Further characterization of XRD showed that BN-PEG has peaks of PEG and BN, and the peak intensity of PEG is much higher than BN in the BN-PEG, showing that degree of coating with PEG is high, so we observed high water solubility in solution of BN-PEG (Fig. 2). The TGA of BN-PEG was shown in Fig. 3; the losing weight of PEG was presented at 412 °C, but because BN was very stable in high temperature or in flames, and so the losing weight of BN was only about 3.74 % until 800 °C. Compared to the TGA results of single PEG and BN, the residues represented the BN nanoparticles (~86.58 %) but the losing weight of PEG was (~11.42 %) in BN-PEG (Fig. 3), which may be owing to scale screening of BN-PEG. In this work, the bigger scale nanoparticles of BN-PEG were removed by centrifugal separation, so we selected the small scale nanoparticles of BN with PEG (Fig. 1). However, high content of PEG in small size of BN-PEG would also exist high solubility and biological compatibility (Fig. 4), but which causes the characteristic absorption bands of BN in BN-PEG can hardly be observed by XRD (Fig. 2).

### Biodistribution of BN-PEG Through Labeling of ^99m^Tc

**Fig. 5:**
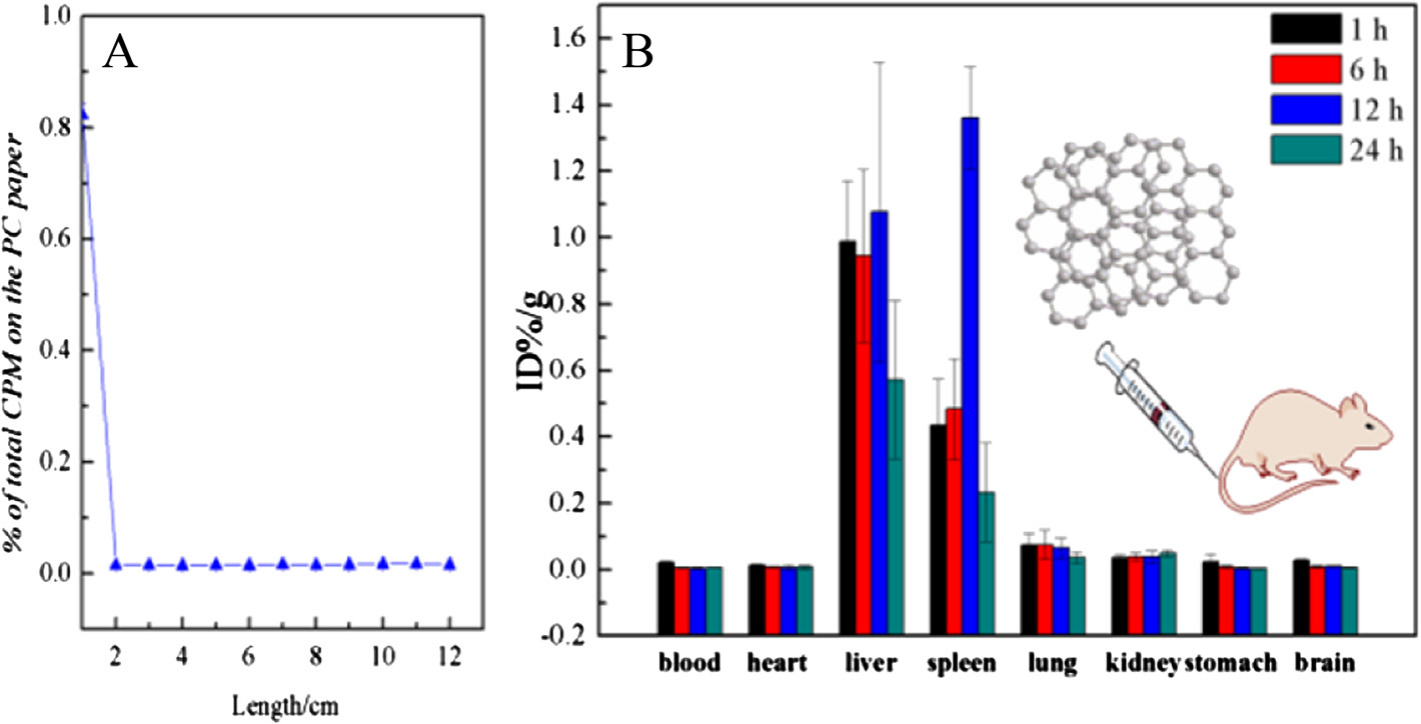
The labeling yields of ^99m^Tc-BN-PEG were measured by paper chromatography (**a**); and their biodistribution in the blood, heart, liver, spleen, lung, kidney, stomach, and brain of mice at 1, 6, 12, and 24 h after exposure to ^99m^Tc-BN-PEG (**b**) (±sem, *n* = 6)

BN is insoluble in water, but it would be dissolved easily in water after coating with PEG. The PEG contained many hydroxy groups, so the unfilled electron orbits of Tc(V) were filled immediately by electrons donated by four hydroxy groups from BN-PEG. Stable chelate compound ^99m^Tc-BN-PEGs were formed with high labeling rate (Fig. 5a). It was reported that, after single exposure to Na^99m^TcO_4_, most are cleared quickly by urine without obvious retention in the organs [18]. However, in this study, we observed that ^99m^Tc-BN-PEG was highly distributed in the liver, spleen, and other organs of mice in 24 h after injection. These results suggest that the labeling compounds have tracer properties and can effectively respond to the behavior of BN in vivo. The radiolabeling yields of ^99m^Tc-BN-PEG were determined by paper chromatography, with normal saline as the solvent (Fig. 5a). The radiolabeling yields were over 90 %.

The Fig. 5b showed that BN-PEG was mainly distributed in the liver and spleen in 24 h but with lower distribution in the lung, interestingly, which is different with the biodistribution of other carbon nanoparticles [19, 20]. In our previous works [21–23], we reported that the distribution in the lung and stomach is the highest for the graphene oxide (GO), carbon nanotubes, and nano-diamond mainly. However, the distribution of BN-PEG has a good agreement with graphene oxide coated by PEG [24]; the BN and GO coated by PEG mainly accumulated in the reticuloendothelial system (RES) such as the liver and spleen (Fig. 5b), meaning that the coating of PEG affected and changed the behavior and fate of the nanoparticles in vivo. The high distribution of BN/GO-PEG in macrophage organs could be attributed to Kupffer cells which could devour the nanoparticles so as to decrease the tissues toxicity, but the high accumulation in the lung is owing to capture role of the pulmonary capillary bed on nanoparticles [18]. After coating with PEG, the nanoparticles containing BN and graphene would pass through the pulmonary capillary bed, and entered into the liver and spleen with the development of the circulation of the blood; in here, most of them would be devoured by the Kupffer cells. The finding of the processes would be useful and important for us to protect and cure worker from harm of BN, and it would provide some new route to design the drug delivery system of BN.

Moreover, the peak of biodistribution of BN-PEG is at about 16 h post injections (Fig. 5b), but which is inconsistent with the dynamics of GOs (~2 h) [23], and this may be due to the structure difference of BN and GOs. We also found lower distribution of BN-PEG in the kidney and brain tissues (Fig. 5b). The kidney is the excretory system of nanoparticles through urine, and some papers have reported that the nanoparticles can be excreted by the urine with high biodistribution in the kidney, showing that the BN-PEG could be excreted with development of time, so the distribution in kidney increased slowly and reached the peak at 24 h (Fig. 5b). The biodistribution of BN-PEG in the brain was first reported for most of the nanoparticles in this work. It is generally accepted that the carbon nanoparticles and metal nanomaterials could not pass through blood-brain barrier, so it is very hard to deliver drug with these nanoparticles [25–27]. However, we found a degree of enrichment in brain with slow elimination, so we conferred that the BN-PEG would be able to pass through the blood-brain barrier. The result would be valuable to design the drug delivery system of BN-PEG for curing the disease in brain.

### Histopathology

**Fig. 6:**
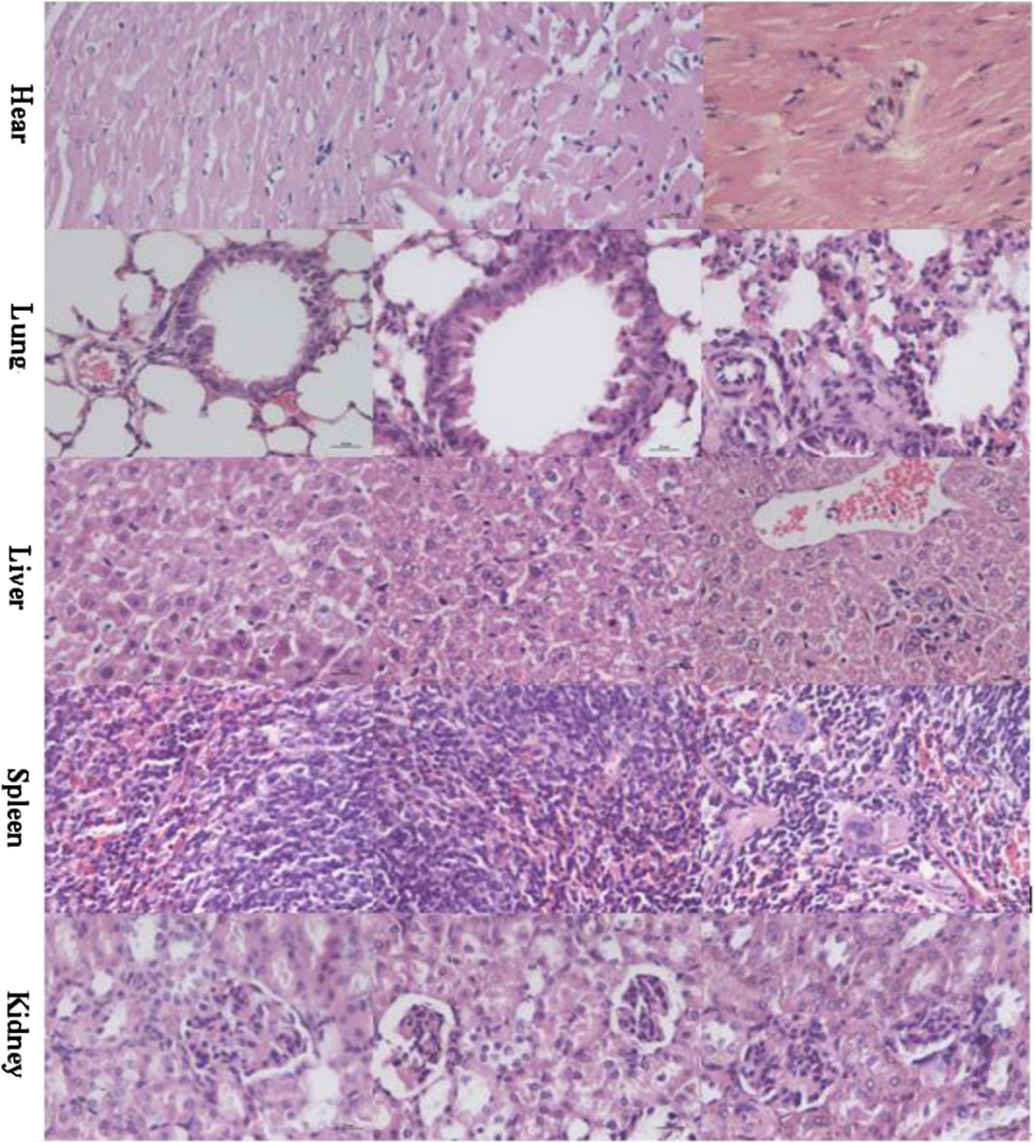
The tissue histology sections after exposure to normal saline, PEG, and BN-PEG to mice

The sections of organic tissues were cut to perform microscopic examination, and shown in Fig. 6. The results showed that the BN-PEG nanoparticles could cause the extensive injury on tissues contained from the liver, spleen, kidney, lung, and heart, and the control groups of normal saline and single PEG are normal. The cell lineage disorder, hepatic lobules disappeared, and hydropic degeneration with focal inflammation could be observed from the liver section. The splenic sinus eclasis, size disorder of follicular, hyperplasia of extramedullary hematopoietic giant cells and small vessels could be seen from the spleen section. Alveolar wall thickening, epithelial cell proliferation, intraluminal secretions, bronchial epithelial disorder, and proliferation of interstitial small blood vessels could be found in lung tissues. The lesion of the kidney could also be observed with glomerular swelling, smaller glomerular capsular, mesangial cell proliferation. Interestingly, we could also observe the severe lesion from pathological section of the heart tissue, although muscle fibers are still arranged neatly, slightly striated muscle cell proliferation with increased cell nucleus. As can be seen from these results, the toxicity of BN-PEG is very strong, the toxicity of nanoparticles on the lung, liver, spleen, and kidney were reported in many papers [21–23]. However, the striated muscle cell proliferation and the increased cell nucleus would result from myocardial hypertrophy, further inducing heart failure. Abdelhalim M.A.K [28] reported that the gold nanoparticles (~200 nm) (GNP) could induce the heart injury, and they thought that the pathological changes may be the results from GNPs interference with the antioxidant defense mechanism. While they also conferred that the pathological changes would be more obvious for the smaller size of nanoparticles, which would cause significantly greater oxidative stress. In this work, in comparison with graphene, although their thickness is single atom layer, the size of BN-PEG is very small (10–20 nm), so it would led to a stronger oxidative stress so as to induce lesions in the heart. Moreover, the BN-PEG nanoparticles could also be observed clearly in section of some tissues contained from the heart and kidney, interestingly, it could not be found obviously in the lung and liver with high distribution. The carbon nanoparticles such as graphene oxides, fullerenes, and carbon nanotubes were seen in the lung, liver, and spleen clearly; our previous papers referred that a few nanoparticles of graphene oxides could be observed in the kidney, which is owing to the excretion of nanoparticles through urine [22, 23]. The size of BN-PEG nanoparticles were much smaller than the graphene oxides. Next, these tissue lesions would be studied deeply through the test of biochemical indicators.

### The Biochemical Characteristics in Serum

**Fig. 7:**
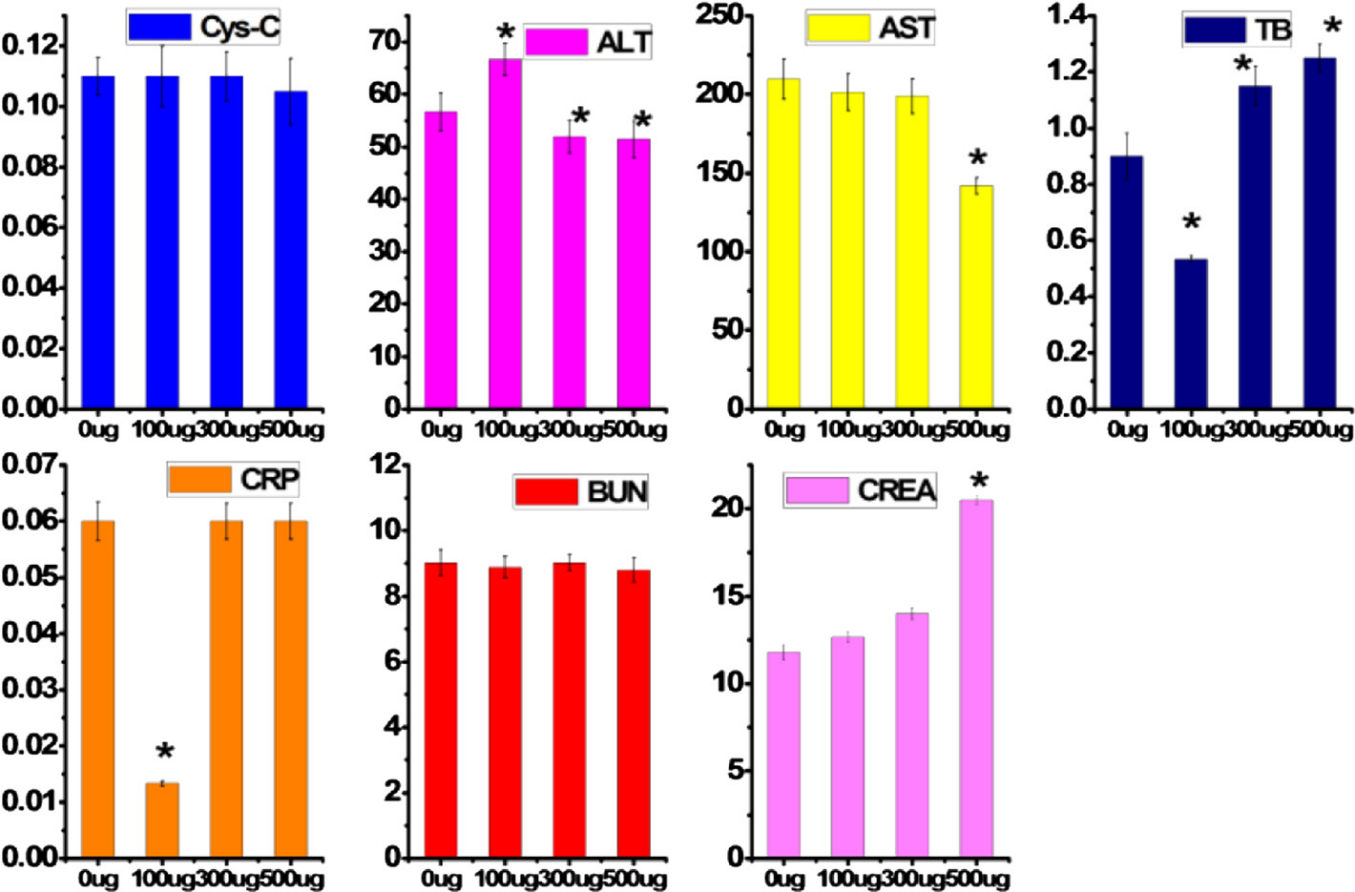
Effect of BN-PEG injection dose on biochemistry index contents in serum in vivo in mice. *p < 0.05 for groups vs. control group (0ug); ±sem. *n* = 6

To reveal any potential toxic effect of BN-PEG on the liver and heart of the treated mice, we also carried out the test of blood biochemistry. Mice injected respectively with the mixed solution of normal saline, and 0, 300, and 500 μg BN-PEG were sacrificed at 24 h for blood collection (six mice per group). Various biochemistry parameters were tested with particular attention paid to the liver and kidney function markers including cystatin C (Cys-C), creatinine (CREA), alanine aminotransferase (ALT), aspartate transaminase (AST), total bilirubin (TB), C-reactive protein (CRP), and blood urea nitrogen (BUN), then these parameters would be compared with control groups (0 μg) using SPSS software, *p* < 0.05 denotes a significant difference, all results were shown in the Fig. 7. We cannot find the significant difference of BUN and Cys-C between exposure and control groups, because BUN and Cys-C are sensitive to the physiological function of kidney. So, this may suggest that the exposure of BN-PEG did not damage the kidney functions. However, TB is also an index for reflecting the change of kidney function, as compared with normal groups; we found that TB content decreased significantly by exposure of 100 μg/mouse BN-PEG, increased significantly by exposure of 300 and 500 μg/mouse BN-PEG, suggesting that to some degree, the kidney function can be affected by exposure to BN-PEG. ALT, AST, CREA, and TB are sensitive to the liver function; Fig. 7 shows that contents in serum of AST, ALT, and CREA can be significantly changed after injection with 100 or 500 μg/mouse BN-PEG, and meaning that injection of BN-PEG can lead to abnormal liver function, which is consistent with other references. Interestingly, we have found high accumulation in heart, which may indicate that BN-PEG would damage heart tissue, so, we also test the CRP in serum as a sensitive index to the heart. As compared with control group, the content of CRP can be decreased by the exposure of 100 μg/mouse BN-PEG, with the increase of injection doses, the content of CRP in serum would return back to normal level. This indicates that the BN-PEG with certain dose may damage the heart function as a result of high biodistribution of heart, but detailed mechanism needs further study in the future. In a word, although the covering of PEG on surface of BN can improve the water solubility and biocompatibility; exposure of high-dose BN-PEG is still very dangerous for animals in vivo.

## Conclusions

The BN is a poorly water-soluble nanoparticle, so the data of its in vivo biodistribution and safety is unclear up to now. Therefore, the highly soluble BN-PEG was prepared by an improvement of existing methods, and we, for the first time studied in vivo biodistribution and biotoxicity of BN-PEG by a radiolabeling method in mice. Both biodistribution measurements based on ^99m^Tc labeling suggest that the BN-PEG mainly distributed in the liver, spleen, and lung, and observations from histological slices show that it could cause obvious tissue lesions in liver, spleen, lung, and heart. The heart damage is a novel and interesting finding for understanding and treating the toxicity of BN nanoparticles on mice; the test of biochemistry parameters including Cys-C, CREA, ALT, AST, TB, CRP, and BUN in serum further confirms the result. Therefore, the BN-PEG nanoparticles are the hazardous materials in vivo, and its biologic toxicity could be taken seriously.
